# Mast cells and eosinophils in invasive breast carcinoma

**DOI:** 10.1186/1471-2407-7-165

**Published:** 2007-08-29

**Authors:** Rose-Marie Amini, Kirsimari Aaltonen, Heli Nevanlinna, Ricardo Carvalho, Laura Salonen, Päivi Heikkilä, Carl Blomqvist

**Affiliations:** 1 Department of Genetics and Pathology, Uppsala University Hospital, Uppsala, Sweden; 2 Department of Oncology, Helsinki University Central Hospital, Helsinki, Finland; 3 Department of Obstetrics and Gynecology, Helsinki University Central Hospital, Helsinki, Finland; 4 Department of Medicine, Karolinska Institute, Stockholm, Sweden; 5 Department of Pathology, Helsinki University Central Hospital, Helsinki, Finland; 6 Department of Oncology, Radiology and Clinical Immunology, Uppsala University Hospital, Uppsala, Sweden

## Abstract

**Background:**

Inflammatory cells in the tumour stroma has gained increasing interest recently. Thus, we aimed to study the frequency and prognostic impact of stromal mast cells and tumour infiltrating eosinophils in invasive breast carcinomas.

**Methods:**

Tissue microarrays containing 234 cases of invasive breast cancer were prepared and analysed for the presence of stromal mast cells and eosinophils. Tumour infiltrating eosinophils were counted on hematoxylin-eosin slides. Immunostaining for tryptase was done and the total number of mast cells were counted and correlated to the proliferation marker Ki 67, positivity for estrogen and progesterone receptors, clinical parameters and clinical outcome.

**Results:**

Stromal mast cells were found to correlate to low grade tumours and estrogen receptor positivity. There was a total lack of eosinophils in breast cancer tumours.

**Conclusion:**

A high number of mast cells in the tumours correlated to low-grade tumours and estrogen receptor positivity. Eosinophils are not tumour infiltrating in breast cancers.

## Background

The stroma surrounding the malignant cells is important for the growth and spread of the malignant tumour. Recently, accumulating evidence suggests that the local inflammatory process, previously believed to be the host response against cancer, might actually contribute to the development of malignancy and the inflammatory response in the tumours has gained increased attention [1-4]. In follicular lymphomas, gene expression analyses have shown that genes associated with infiltrating inflammatory cells are more important than the tumour cells themselves for predicting outcome [5].

Tumour-associated eosinophilia has been observed in human cancers, sometimes with different results regarding their association with clinical outcome [6-11].

Mast cells derive from a specific bone marrow progenitor cell and migrate into tissues where they mature depending on the microenvironmental conditions.

Mast cells may promote tumour development through many different ways. Mast cells could facilitate tumour angiogenesis through heparin-like molecules and heparin could further permit neovascularisation and metastases through its anti-clotting effects [12]. Moreover, mast cells secrete histamine and growth factors, such as VEGF (vascular endothelial growth factor), PDGF (platelet derived growth factor), SCF (stem cell factor) and NGF (nerve growth factor). Mast cells are also rich in metalloproteases that contribute to the majority of proteolytic components necessary for tumour invasiveness [3]. On the other hand, mast cells could also be detrimental to tumour growth by secreting several cytokines and proteolytic enzymes participating in inducing apoptosis of the malignant cells, such as IL-4 [13]. The dual role of mast cells in inhibiting or promoting tumour growth needs to be further investigated [14]. Few studies of stromal mast cells in invasive breast carcinomas have been done and two previous studies have indicated that many stromal mast cells are correlated to a favourable prognosis [15-17]. Furthermore, in lymph nodes of women with breast cancer a higher number of mast cells were found in the non-involved axillary lymph nodes in those women with a better prognosis [18]. In women with axillary lymph node metastasis more mast cells were found in the non-involved axillary lymph nodes [19]. These findings might indicate a protective effect of mast cells, possibly exerting a cytotoxic effect on the tumour cells. However, the studies are still few and further investigations are needed in order to elucidate the precise role of mast cells in the tumourigenesis. Mast cells interact with eosinophils in bidirectional interactions and cytokine networks [20]. Eosinophils may serve as a source of SCF which induces the growth of mast cells regulating their activation, degranulation and chemotaxis.

In Hodgkin lymphomas, many eosinophils in the tumours are correlated to an unfavourable prognosis [7] and many mast cells have been associated with an adverse clinical outcome [21]. Interactions between mast cells and eosinophils in Hodgkin lymphomas are attributed to the CD30L-CD30 interaction [22].

Tumour infiltrating eosinophils have previously been investigated in colon carcinomas [6,8] but little data is known about breast cancer. In colon cancer a high amount of stromal eosinophils have correlated to a better prognosis.

The aim of this study was to determine if the presence and amount of tumour infiltrating mast cells and eosinophils in breast cancer correlated to prognosis.

## Methods

The patients were newly diagnosed breast cancer patients collected in 1997–1998 at the Helsinki University Hospital as described previously [23]. Tissue microarray (TMA) blocks were made from 234 cases of invasive breast cancer as described [24,25]. For tissue microarray construction, a hematoxylin and eosin stained section was made from each paraffin block to define a representative tumour region. The most representative area of the tumours was punched to produce the TMA blocks including four cores (diameter 0.6 mm) from each of the original blocks.

### Immunohistochemistry

The formalin-fixed paraffin-embedded tissue material was cut in 3–4 μm thick sections and deparaffinised. Immunostainings for tryptase diluted 1:4000 (Chemicon International) (mast cell specific, indicates the presence of all mast cells both intact and degranulated mast cells in tissues) were performed with an automatic immunostainer (Ventana, Tucson AZ) at 37° using the avidin-biotin peroxidase complex and AEC procedures [26]. The peroxidase was developed using the DAB technique. Primary antibody was diluted 1:4000. Immunostaining results for Ki 67, estrogen receptor (ER) and progesterone receptor (PR) were collected from the pathology reports for all the tumours. As negative control, only buffer and secondary antibody was applied.

### Mast cells and eosinophil counting

Mast cells and eosinophils were counted in one high-power field (40× objective) in each tissue core and the mean value of the cells present was estimated.

### Statistical methods

All statistical analyses were made utilising Macintosh SPSS 11 statistical software package. The association between mast cells, proliferation marker Ki 67, PR and ER was tested by computing the nonparametric Spearman correlation coefficient. Mast cells were included as a continous variable as the mean number of the counted fields. Survival analysis with mast cell count as a continous variable were performed with the Cox proportional hazard model.

### Ethics

This study was approved by the Ethical Committee at Helsinki University Hospital

## Results

Two-hundred and thirty-four women were included in the study. The median age was 54 years (range 27–95). Tumour characteristics and staging is shown in table 1. Patients were treated according to the recommended guidelines in Helsinki University Hospital at that time.

**Table 1:** Clinical and histological characteristics of all the patients with high (>mean 7.3) or low (<7.3) mast cell count.

**Characteristics**	**Number of patients (%)**	**Mast cell count high**	**Mast cell count low**	**p-value**
**Tumour stage (T)**				0.462
1	129 (55)	69	60	
2	89 (38)	45	44	
3	7 (3)	4	3	
4	7 (3)	5	2	
not known	2 (1)	1	1	
**Axillary lymph node metastasis (N)**				0.055
Present	118 (50)	66	52	
Absent	114 (49)	58	56	
Not known	2 (1)	0	2	
**Distant metastasis (M) at the time of diagnosis**				0.656
Absent	208 (89)	110	98	
Present	11 (5)	7	4	
Not known	15 (6)	7	8	
**Histology**				0.927
Ductal	163 (70)	80	83	
Lobular	41 (17)	22	19	
Mixed	26 (11)	20	6	
Mucinous	3 (1)	2	1	
Papillary	1 (1)	1	0	
**Elston-grading**				0.001
1	65 (28)	44	21	
2	98 (42)	55	43	
3	71 (30)	25	46	
**Estrogenreceptorstatus**				<0.0005
Positive	179 (76)	105	74	
Negative	44 (19)	15	29	
Not known	11 (5)	4	7	
**Progesteronreceptorstatus**				0.006
Positive	159 (68)	95	64	
Negative	65 (28)	26	39	
Not known	10 (4)	3	7	

In brief, all patients except one underwent surgery, mastectomy in 121 and sector resection in 112. Postoperative radiotherapy was given to 204 and adjuvant chemotherapy to 92 patients. The chemotherapy regimen was CMF (cyclophosphamide, methotrexate and 5-fluorouracil) in 83 patients or FEC (cyclophosphamide, epirubicine and 5-fluorouracil) in 6 patients and three patients had other chemotherapy regimen. Adjuvant endocrine therapy with either tamoxifen (n = 82) or toremifen (n = 20) was given to 102 patients.

Grading was done according to Elston-Ellis [27]. Sixty-five (28%) were grade 1, 98 (42%) were grade 2 and 71 (30%) were grade 3. (Table 1).

Twenty-two patients relapsed locally (localisation: chest 14 patients, axilla 5 patients and clavicular lymph nodes 3 patients). 58 patients developed distant metastasis. The median follow-up time of surviving patients was 62 months (range 17–96).

**Figure 1:**
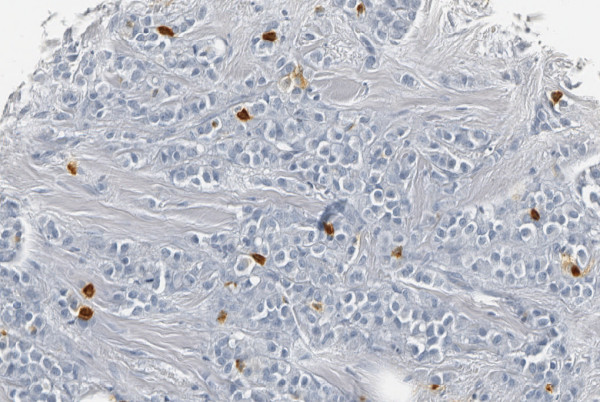
Mast cells express a dark brown immunoreactivity in the cytoplasm.

Mast cells were found mainly in the tumour stroma adjacent to the neoplastic cells (fig 1) but in some cases mast cells infiltrated within the islands of tumour cells.

**Figure 2:**
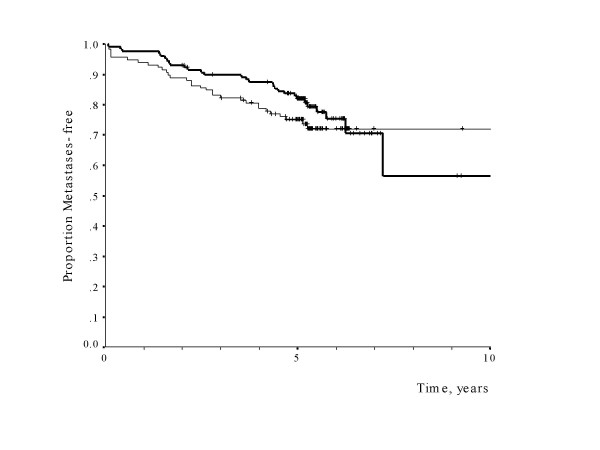
Metastases-free survival of patients with high mast cell count (>median 7.3) in bold line compared to those with low mast cell count (<7.3).

Mean mast cell count was 7.3 (range 0–59). Eosinophils were not present at all in any tumour. Mast cells correlated significantly to low grade tumours (p = 0.001) and ER positivity (p < 0.0005). There was no correlation to the proliferation marker Ki 67. Mean mast cell count had no significant impact on metastasis-free survival (p = 0.133, relative hazard for high-versus low-mast 0.67, 95% confidence level 0.40 – 1.13) (fig 2), overall survival (p = 0.17, relative hazard 0.66, 95% CI 0.37–1.20) or local recurrence (p = 0.73, relative hazard 0.86, 95% CI 0.36–2.05).

## Discussion

Identifying new prognostic markers for patients with node-negative breast cancers is an important issue since the current treatment guidelines recommend adjuvant treatment for about 90 percent of the patients with node-negative disease, even though only about 30 percent of the patients actually will benefit from the therapy. Thus, it is important to identify new markers for this subgroup of patients who might then not need adjuvant chemotherapy.

The presence of mast cells was associated to low tumour grade and estrogenreceptor positivity. These are factors that are associated with a favourable prognosis in breast cancer. This might, thus be interpreted as that many mast cells is an additive favourable prognostic sign even though it was not an independent prognostic marker in this study. Eosinophils were not present at all in any of the tumour samples.

Although the mast cell count did not have a statistical impact on disease-free or overall survival in this study, this may well be due to the small number of patients. A non-significant trend towards both an improved local and distant tumour control was seen in patients with high mast cell counts, as well as an improved survival. The magnitude of this putative prognostic impact, however, seemed to be smaller than with classical clinical prognostic factors like nodal status and tumour size [28].

Mast cells accumulate around tumours and could either promote or inhibit tumour growth depending on the local stromal conditions. The presence of stromal mast cells in other malignant tumours have been of interest, but comprehensive studies are few. In colon cancer, a high amount of mast cells have been associated with lower rates of lymph node metastasis and distant metastasis [29] and many eosinophils have been associated with a better prognosis [6]. In lung cancers, a higher mast cell count has been correlated to tumour progression [30] and also correlated to microvessel density [31] in some studies, but in another study there was a lack of correlation between mast cells, eosinophils and microvessels [32]. In squamous cell carcinomas of the esophagus [33] and cervix [34] a high number of mast cells in the tumours were likewise associated to both microvessel density and tumour progression. Similarly, in malignant melanomas [35] and Hodgkin lymphomas [21] mast cells have been related to an adverse clinical outcome. The finding of many stromal mast cells close to microvessel and the association to microvessel density might be explained by contribution of mast cells to neo-angiogenesis in these tumours. However, our finding of the correlation of large number of mast cells to favourable prognostic factors in breast cancer indicate that other factors must be taken into consideration. Thus, the presence of stromal mast cells in different tumours and their precise role in the tumourigenesis needs to be further investigated in order to elucidate the different mechanisms behind.

The absence of eosinophils indicates that other mechanisms than the mast cell/eosinphil axis are involved, or might rather indicate another composition of the inflammatory infiltrate in breast cancers.

## Conclusion

The presence of stromal mast cells in breast cancer correlate to low grade tumours and estrogen receptor positivity. The role of stromal mast cells for prognostic significance in breast cancer warrants further studies. Eosinophils are not tumour infiltrating in breast cancers.

## Competing interests

The author(s) declare that they have no competing interests.

## Authors' contributions

R-M A interpreted and analysed data. KA analysed and interpreted clinical data. RC carried out immunohistochemical analyses. HN, LS and PH contributed with collection and assembly of data and with the TMA construction. CB performed the statistical analyses, interpreted the data and revised the manuscript.

## Pre-publication history

The pre-publication history for this paper can be accessed here:


